# Purification, Partial Characterization and Immobilization of a Mannose-Specific Lectin from Seeds of *Dioclea lasiophylla* Mart

**DOI:** 10.3390/molecules180910857

**Published:** 2013-09-04

**Authors:** Vanir Reis Pinto Júnior, Mayara Queiroz de Santiago, Vinícius José da Silva Osterne, Jorge Luis Almeida Correia, Francisco Nascimento Pereira Júnior, João Batista Cajazeiras, Mayron Alves de Vasconcelos, Edson Holanda Teixeira, Antônia Sâmia Fernandes do Nascimento, Thaiz Batista Azevedo Rangel Miguel, Emilio de Castro Miguel, Alexandre Holanda Sampaio, Kyria Santiago do Nascimento, Celso Shiniti Nagano, Benildo Sousa Cavada

**Affiliations:** 1 Laboratory of Biologically Active Molecules (Biomol-Lab), Department of Biochemistry and Molecular Biology, Federal University of Ceará, Av. Humberto Monte s/n, Bloco 907, Lab. 1075, Campus do Pici, Fortaleza-CE 60440-970, Brazil; E-Mails: juniorreis4@hotmail.com (V.R.P.J.); mayara_delonge@hotmail.com (M.Q.S.); vinnyosterne@gmail.com (V.J.S.O.); jorge.biomol@gmail.com (J.L.A.C.); fnpjunior@yahoo.com.br (F.N.P.J.); jcajazeiras@gmail.com (J.B.C.); mayronvasconcelos@gmail.com (M.A.V.); asamiaf@gmail.com (A.S.F.N.); thaizrangel@gmail.com (T.B.A.R.M.); emiliocmiguel@yahoo.com.br (E.C.M.); kyriasantiago@gmail.com (K.S.N.); 2 Integrated Laboratory of Biomolecules (LIBS), Federal University of Ceará, Department of Pathology and Legal Medicine, Faculty of Medicine, Fortaleza, CE 60430-160, Brazil; E-Mail: edson@ufc.br; 3 Laboratory of Mass Spectrometry Applied to Proteins (LEMAP), Federal University of Ceará, Av. Humberto Monte s/n, Bloco 825, Campus do Pici, Fortaleza-CE 60440-970, Brazil; E-Mails: alexholandasampaio@gmail.com (A.H.S.); naganocs@gmail.com (C.S.N.)

**Keywords:** lectin, *Dioclea lasiophylla*, Diocleinae, toxicity, immobilization

## Abstract

Lectin from the seeds of *Dioclea lasiophylla* (DlyL) was purified in a single step by affinity chromatography on a Sephadex^®^ G-50 column. DlyL strongly agglutinated rabbit erythrocytes and was inhibited by monosaccharides (_D_-mannose and α-methyl-d-mannoside) and glycoproteins (ovalbumin and fetuin). Similar to other Diocleinae lectins, DlyL has three chains, α, β and γ, with mass of 25,569 ± 2, 12,998 ± 1 and 12,588 ± 1 Da, respectively, and has no disulfide bonds. The hemagglutinating activity of DlyL was optimal in pH 8.0, stable at a temperature of 70 °C and decreased in EDTA solution, indicating that lectin activity is dependent on divalent metals. DlyL exhibited low toxicity on Artemia sp. nauplii, but this effect was dependent on the concentration of lectin in solution. DlyL immobilized on cyanogen bromide-activated Sepharose^®^ 4B bound 0.917 mg of ovalbumin per cycle, showing the ability to become a tool for glycoproteomics studies.

## 1. Introduction

Lectins currently represent an important tool in glycobiology studies because of their ability to decipher glycocodes. These proteins/glycoproteins, which have at least one noncatalytic domain of recognition and reversible binding to specific carbohydrates [1], participate in a variety of cellular processes without changing the mono/oligosaccharides involved [2]. These lectins can be employed in a range of biomedical studies, including cancer and immunological research, isolation and characterization of glycoconjugates, and blood typing. In glycoproteomics, studies are facilitated by the large number of natural lectins that recognize and bind to carbohydrates. When immobilized on inert matrices, these lectins are used in affinity chromatography, assisting in the purification and separation of glycoproteins for analytical testing [3].

Recent studies have shown that these plant lectins have several applications inside the body, such as defense [4], or outside the body, such as drug delivery [5], as well as a variety of diagnostic applications for a broad spectrum of diseases [6].

Legume lectins are normally composed of two or four monomers, presenting a molecular mass of about 25–30 kDa, with each monomer presenting a unique, highly conserved carbohydrate binding site, as well as conserved metal binding sites for divalent cations (calcium and manganese). The monomers are associated by noncovalent interactions [7].

*Dioclea lasiophylla* belongs to the family Leguminosae (Fabaceae), tribe Phaseoleae, subtribe Diocleinae. It is endemic in Brazil and found mainly in the northeast region. Diocleinae lectins are a well-studied group of closely related lectins among the leguminous group. Different biological effects associated with these proteins have been described, such as histamine release from rat peritoneal mast cells [8] and anti- and pro-edematogenic effects [9,10]. Minor differences in the ratios of dimeric and tetrameric forms in the lectins and/or differences in the relative orientations of the carbohydrate-binding sites in the quaternary structures have been hypothesized to contribute to the differences in biological activities exhibited by Diocleinae lectins [11]. Such properties make these lectins valuable biomedical tools, and, as such, the characterization and sequence analysis of different lectins belonging to this subtribe are ongoing. Furthermore, Diocleinae lectins provide an excellent system to study the dramatic effects of minor structural differences on functional properties in proteins [12]. The present study aims to purify and characterize the lectin from *D. lasiophylla* seeds, determine its toxicity against *Artemia* sp. and its possible biomedical applications.

## 2. Results and Discussion

The crude extract of seeds of *D. lasiophylla* showed strong agglutination activity in native rabbit erythrocytes treated with trypsin and papain (data not shown). The inhibition assay of hemagglutinating activity with carbohydrates showed that the lectin has specificity for D-mannose and α-methyl-D-mannoside, although activity was not inhibited by _D_-glucose (Table 1). The hemagglutinating activity of DlyL was also inhibited by two glycoproteins, ovalbumin and fetuin, having mannose as their glycan structure.

**Table 1:** Inhibitory effect of saccharides and glycoproteins on the hemagglutinating activity of *Dioclea lasiophylla* lectin.

Carbohydrate	MIC *
d-Glucose	NI **
d-Mannose	25 mM
d-Galactose	NI **
N-acetyl-d-glucosamine	NI **
α-methyl-d-mannoside	6.25 mM
α-methyl-d-galactoside	NI **
α-Lactose	NI **
β-Lactose	NI **
Glycoprotein	
Ovalbumin	0.0625 mg/mL
Fetuin	0.125 mg/mL

* MIC, Minimum inhibitory concentration; ** NI, sugar not inhibitory until a concentration of 100 mM.

**Figure 1:**
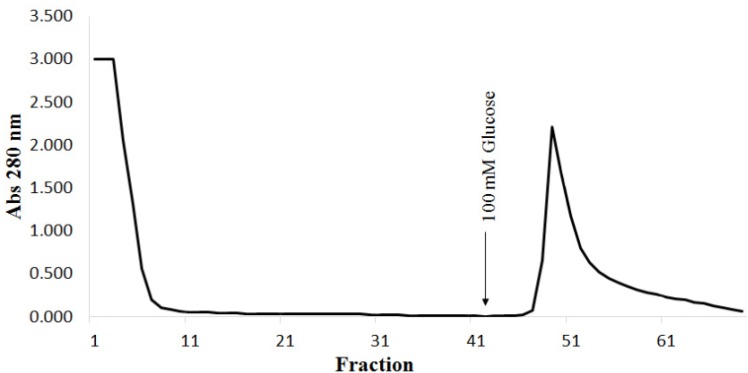
Elution profile of the Sephadex^®^ G-50 affinity chromatography. Approximately 10 mL of the crude extract was applied to the column (6.5 × 1.8 cm) which was equilibrated with 150 mM NaCl plus 5 mM CaCl_2_ and 5 mM MnCl_2_. The lectin was eluted with 100 mM D-glucose in the equilibrium solution described earlier at a flow rate of 1 mL/min. Fractions of approximately 3 mL were collected and monitored for protein content by measuring the absorbance at 280 nm.

Although the monosaccharide d-glucose was unable to inhibit the hemagglutinating activity, the DlyL was purified by a single-step affinity chromatography in Sephadex^®^ G-50 column, a Dextran chromatographic support, on which the lectin was quantitatively retained in the gel and eluted with 100 mM d-glucose, providing strong evidence for carbohydrate-binding properties (Figure 1). The soluble protein content and the specific activity of the crude lectin extract were 5.2 mg/mL and 196 HU/mg proteins, respectively. For the purified lectin, the values were 0.76 mg/mL and 5,390 HU/mg proteins, respectively. The specific activity increased by 27.5-fold in the pure lectin (Table 2).

**Table 2:** Purification of lectin from *Dioclea lasiophylla* seeds.

Fraction	^a^ Total protein (mg/mL)	^b^ Total HU	^c^ Specific activity (HU/mg)	Purification (fold)
Crude extract	5.2	2^10^	196	1
PII (Sephadex^®^ G-50)	0.76	2^12^	5390	27.5

^a^ Protein content; ^b^ Hemagglutinating activity expressed in hemagglutinating units (HU); ^c^ Specific activity calculated as the ratio between hemagglutinating activity and protein content.

**Figure 2:**
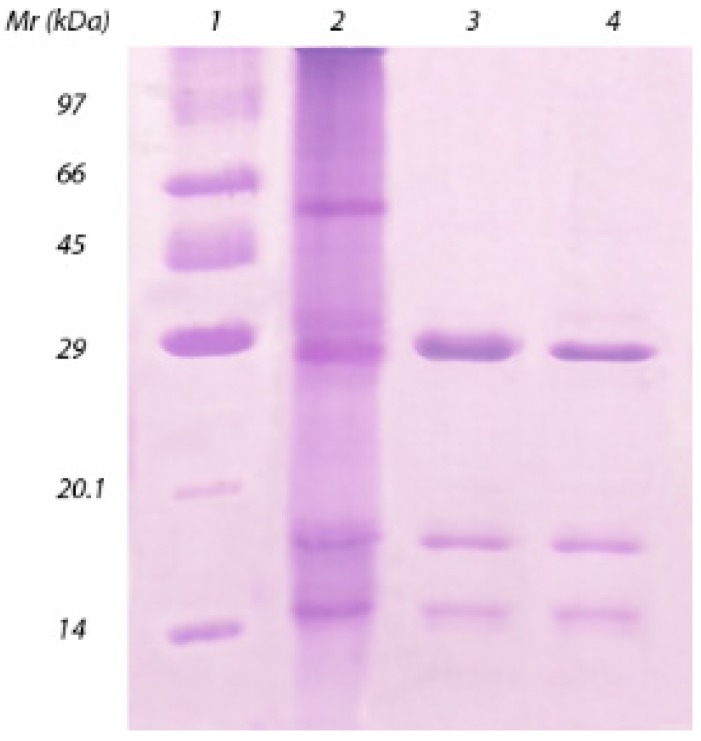
SDS-PAGE. Lane 1: molecular mass markers (phosphorylase b, 97 kDa; bovine serum albumin, 66 kDa; ovalbumin, 45 kDa; carbonic anhydrase, 29 kDa; trypsin inhibitor, 20.1 kDa; and α-lactalbumin, 14 kDa); lane 2: crude extract; lane 3: DlyL (30 µg); lane 4: DlyL (30 µg) with 2% β-mercaptoethanol.

As shown in Figure 2, SDS-PAGE assay of DlyL showed the appearance of three polypeptide chains, α, β and γ. These findings were confirmed by ESI mass spectrometry analysis proving the existence of just one lectin, composed of three chains (α, β and γ). The α-chain has a molecular mass of 25,569 ± 2 Da. The mass of each fragment is 12,998 ± 1 Da (β-chain) and 12,588 ± 1 Da (γ-chain).

The addition of β-mercaptoethanol did not change the electrophoretic profile of DlyL, suggesting the absence of disulfide bonds. DlyL showed no staining by periodic acid-Schiff, suggesting that it is not a glycoprotein. These characteristics correspond to the pattern of other Diocleinae lectins, such as *Dioclea altissima* [13], *Dioclea rostrata* [14], *Dioclea violacea* [15], *Dioclea grandiflora* [16], *Dioclea guianensis* [17], *Dioclea sclerocarpa* [18] and *Dioclea lasiocarpa* [19].

**Figure 3:**
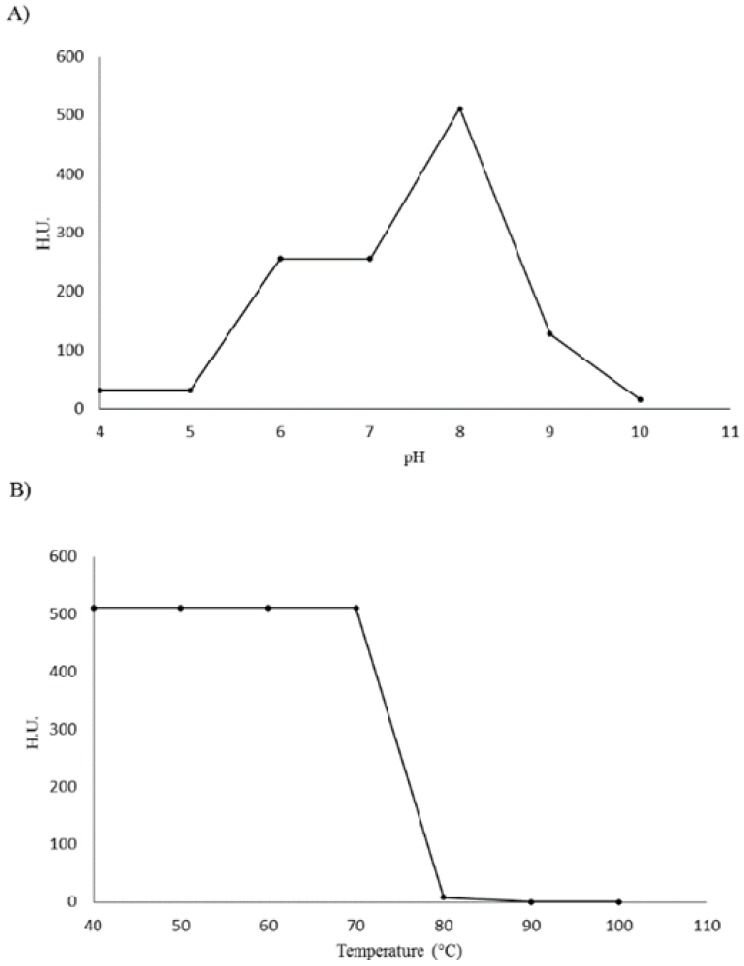
Physicochemical properties from Dioclea lasiophylla lectin. (**A**) Effect of pH on DlyL hemagglutination activity; (**B**) DlyL thermal stability.

DlyL was able to maintain its hemagglutinating activity within a relatively wide pH range with maximal activity observed at pH 8.0 (Figure 3A), indicating that the lectin is more stable in this condition. A further decrease in pH (from 6.0–7.0) reduced the hemagglutinating activity of DlyL by 50%, and in pH 5.0, or lower, lectin activity was negligible. An increase in pH (above 9.0) caused a 75% loss in activity, and in pH 10.0, the lectin was nearly inactivated. DlyL was shown to be quite thermostable, supporting a temperature of 70 °C for 1 h without any loss in its hemagglutination activity (Figure 3B); in 80 °C, the lectin lost 98% of its activity, while at higher temperatures, no agglutination was detected, indicating the thermo inactivation of DlyL. Both pH stability and thermal resistance of DlyL are close to other lectins from the Diocleinae subtribe [18,19,20,21].

**Figure 4:**
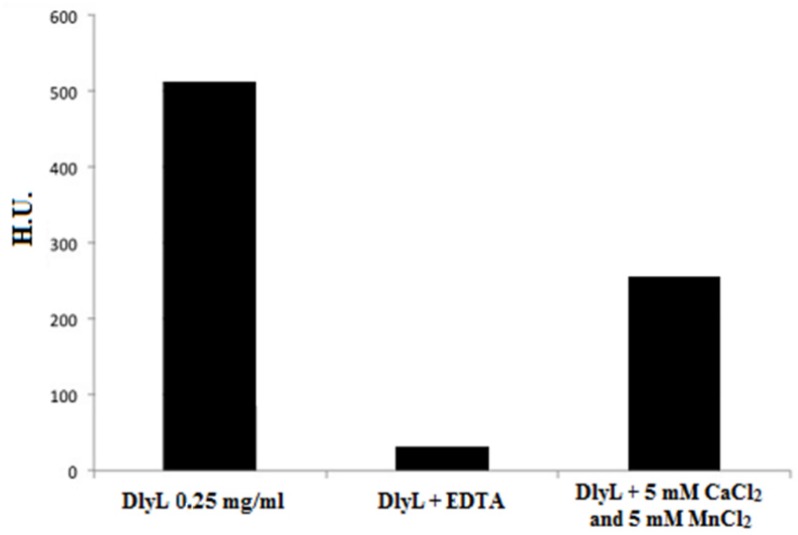
Metal dependence of DlyL. Hemagglutinating activity of native DlyL, demetalized DlyL (dialyzed against 100 mM EDTA, followed by 150 mM NaCl) and metal added to DlyL (5 mM CaCl_2_ and 5 mM MnCl_2_).

After demetalizing the lectin by sequential dialysis against EDTA and NaCl, the hemagglutination activity of DlyL was greatly reduced (loss of 94%). However, the activity was partially recovered after the addition of 5 mM CaCl_2_ and 5 mM MnCl_2_ (Figure 4), showing that DlyL is dependent on these divalent cations for its activity. The requirement for metals is a common characteristic of legume lectins, including several of the Diocleinae subtribe [22].

**Figure 5:**
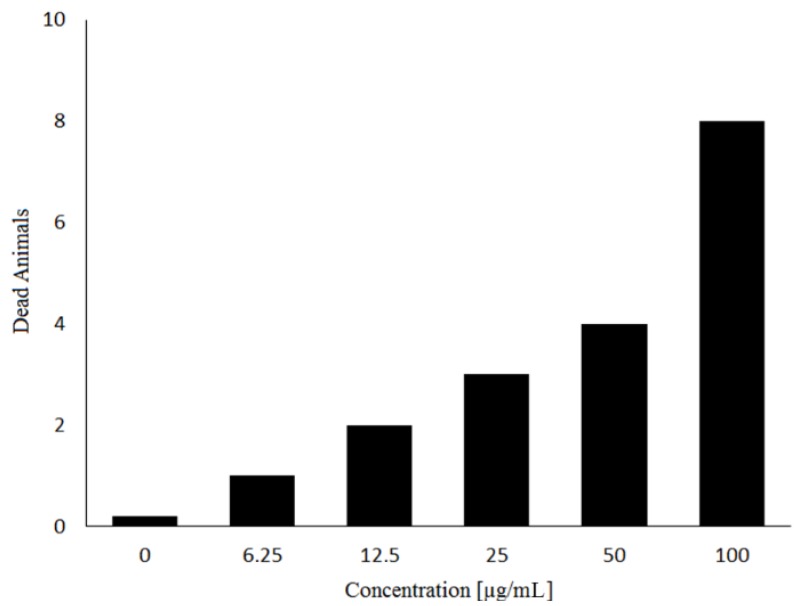
Toxic effect of DlyL at different concentrations on *Artemia* sp. Nauplii.

DlyL has toxic activity against brine shrimp (*Artemia* sp. nauplii), exhibiting LC_50_ value of 45.85 µg/mL. This effect proved to be dose-dependent and was observed in a concentration range from 6.25 to 100 µg/mL (Figure 5). The *Artemia* lethality test [23,24] has been used successfully to determine the toxicity of biological molecules that have a variety of pharmacological activities, including anticancer agents, antivirals, insecticides, pesticides, and anti-HIV compounds [25,26,27]. Previous work showed that other lectins, such as ConA-like, also have toxic effect [28], with LC_50_ between 2.52 and 15.5 µg/mL. In comparison to other similar lectins, DlyL exhibited low toxicity against *Artemia*. 

**Figure 6:**
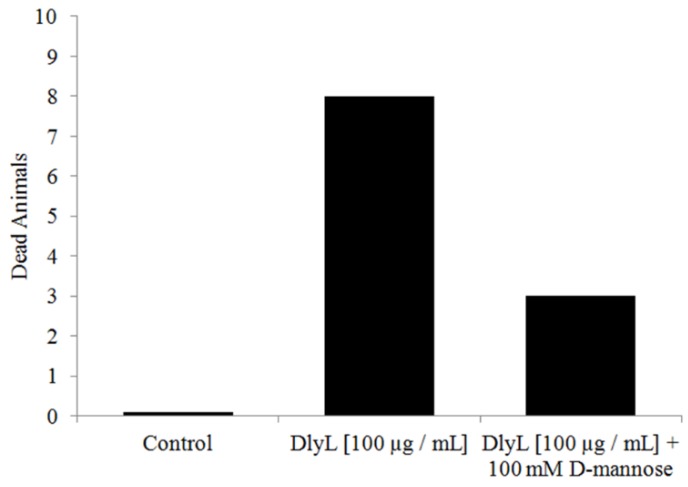
Toxic effect of DlyL (100 µg/mL) and DlyL (100 µg/mL) previously incubated with 100 mM d-mannose.

The lethality of animals decreased (reduction of 62.5%) when the lectin was incubated with d-mannose (Figure 6), showing that the carbohydrate recognition domain is responsible for the toxic activity against *Artemia*.

**Figure 7:**
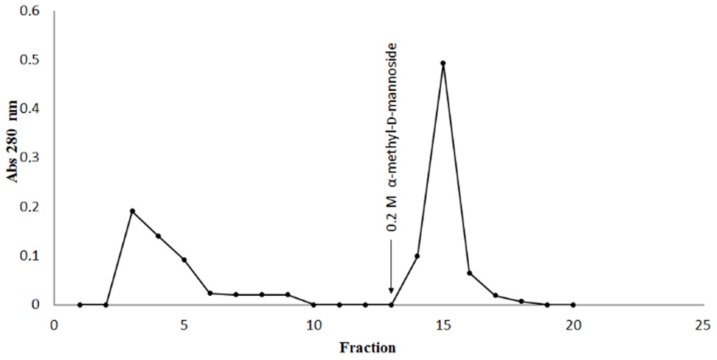
Chromatography on DlyL-Sepharose 4B. The column (0.8 × 2.7 cm) was equilibrated with 150 mM NaCl plus 5 mM CaCl_2_ and 5 mM MnCl_2_. Sample (2 mg) of ovalbumin was applied to the column. The lectin affinity support was then washed with the same buffer. Elution was carried out at 25 °C at a flow rate of 3 mL/min. Arrows demonstrate the points at which eluent (α-methyl-d-mannoside) was added. Fractions of 1.0 mL were collected.

DlyL was successfully immobilized in Sepharose 4B (yield of 97.34%) and was capable of binding strongly to ovalbumin (Figure 7). The glycoprotein was eluted with α-methyl-d-mannoside, a saccharide that best inhibited hemagglutinating activity, showing that the carbohydrate recognition domains remain active after immobilization and are responsible for interaction of DlyL-Sepharose 4B with ovalbumin. DlyL-Sepharose 4B column was able to efficiently isolate glycoprotein (0.917 mg), displaying high potential for glycoproteomics studies.

## 3. Experimental Section

### 3.1. Plant Material

Mature *D. lasiophylla* seeds were collected in Ceará (Brazil) and identified by in the Herbarium Prisco Bezerra EAC in the Department of Biology of the Federal University of Ceará (UFC).

### 3.2. Purification of DlyL

The seeds were ground to a fine powder in a coffee mill. Soluble proteins were extracted in 150 mM NaCl plus 5 mM CaCl_2_ and 5 mM MnCl_2_ [1:10 (w:v)] under constant stirring for 4 h at room temperature. Subsequently, the extract was centrifuged at 10.000 × g at 4 °C for 20 min, and the supernatant was filtered on filter paper (Whatman^TM^). Protein concentration was determined by the method described by Bradford [29], using bovine serum albumin as a standard. The resulting supernatant was applied to Sephadex^®^ G-50 affinity column (6.5 × 1.8 cm) previously equilibrated with extraction solution. Unbound material (P1) was washed with the same solution, and lectin was eluted with 100 mM d-glucose in the equilibrium solution; eluted fractions were monitored by absorbance at 280 nm. The eluate was collected, dialyzed against distilled water, and lyophilized. The homogeneity of the sample was monitored by SDS-PAGE, and the pure protein was used for characterization tests.

### 3.3. Hemagglutination Tests

The hemagglutination assay was performed in microtiter plates by serial dilution using 3% native rabbit erythrocytes treated with the proteolytic enzymes trypsin and papain [30]. Hemagglutinating unit (HU) was expressed as a title (the value of the highest dilution giving a positive hemagglutination) per mL of sample.

### 3.4. Inhibition Assay

The carbohydrate binding specificity of DlyL was determined by the ability of different sugars and glycoproteins to inhibit erythrocyte agglutination, as assessed by minimum inhibitory concentration [31]. Serial dilutions were made of sugar/glycoproteins (initial concentration of 100 mM/1 mg/mL, respectively), including d-glucose, d-mannose, d-galactose, N-acetyl-d-glucosamine, α-methyl-d-mannoside, α-methyl-d-galactoside, α-lactose, β-lactose, chicken egg white albumin (ovalbumin) and fetuin, prepared in 150 mM NaCl. DlyL (32 HU) was added to each dilution.

### 3.5. SDS-PAGE

SDS-PAGE was carried out in accordance with the procedure described by Laemmli [32] using a Mini-Protean II apparatus (BioRad; Milan, Italy) in 0.75 mm vertical gel slabs containing a 12.5% polyacrylamide separation gel and 4% polyacrylamide stacking gel. Samples were dissolved in 0.88 M Tris-HCl (pH 6.8), 2% SDS buffer, 1% bromophenol blue and 12.5% glycerol, followed by heating at 100 °C for 5 min. The electrophoretic run was conducted at constant current of 25 mA for 60 min. The bands were visualized by staining with Coomassie Brilliant Blue R-250. The molecular markers were phosphorylase b (97 kDa), bovine serum albumin (66 kDa); ovalbumin (45 kDa) carbonic anhydrase, (29 kDa); trypsin inhibitor (20.1 kDa) and α-lactalbumin, (14 kDa). The presence of disulfide bonds was determined by adding 2% β-mercaptoetanol in the sample buffer, and the carbohydrate presence on the structure of the DlyL was determined by staining with periodic acid-Schiff according to Zacharius and colleagues [33].

### 3.6. Mass Spectrometry

The averaged molecular mass of DlyL was determined by electrospray ionization mass spectrometry (ESI-MS) using a hybrid quadrupole/ion mobility separator/orthogonal acceleration-time of flight mass spectrometer (Synapt HDMS System, Waters Corp., Milford, MA, USA). The protein solution (10 µmol/µL in 50% acetonitrile and 0.2% formic acid) was infused into the system at a flow rate of 10 µL/min. The capillary voltage and the cone voltage were set at 3.5 kV and 40 V, respectively. The source temperature was maintained at 90 °C, and nitrogen was used as a drying gas (flow rate of 150 L/h). The instrument was calibrated with average m/z (charges +12 to +26) of horse heart myoglobin. The data were acquired with the Mass Lynx software, v. 4.0 (Waters Corp., Milford, MA, USA, 2002). The multiply charged spectra were deconvoluted using maximum entropy techniques [34].

### 3.7. Effect of pH and Temperature on Lectin Activity

In order to determine the stability of the protein at different pH values, purified lectin solution (0.25 mg/mL in 150 mM NaCl) was dialyzed for 24 h against different pH buffers (all containing 150 mM NaCl), ranging from pH 4.0 to 10.0: 100 mM sodium citrate (pH 4.0 and 6.0), 100 mM sodium acetate (pH 5.0), 100 mM sodium phosphate (pH 7.0), 100 mM Tris-HCl (pH 8.0) and glycine-NaOH (pH 9.0 and 10.0). The activity of the lectins after dialysis was measured by hemagglutination activity. The effect of temperature on the activity of DlyL was studied by incubating samples of lectin solution (0.25 mg/mL) at different temperatures (40 °C to 100 °C) for 60 min with increments of 10 °C and measuring the residual activity by hemagglutination tests.

### 3.8. Effect of Divalent Cations

To evaluate the requirement of divalent cations for lectin activity, a sample of lectin solution (0.125 mg/mL) was demetalized by dialysis against 100 mM ethylenediaminetetraacetic acid (EDTA) containing 150 mM NaCl for 24 h; EDTA excess was removed posteriorly with dialysis against 150 mM NaCl. The change in activity was determined after addition of 5 mM CaCl_2_ and 5 mM MnCl_2_. Both the demetalized and metal-added samples had their activity determined via hemagglutination tests.

### 3.9. Artemia Toxicity Tests

Cytotoxicity was studied using *Artemia* nauplii. The *Artemia* cysts were hatched in artificial seawater at 28 °C under constant light and aeration. The cysts were incubated in a glass tube with 1 g cysts per liter of artificial seawater. After a period of 48 h, the aeration was halted, and the light was directed to the bottom of the tube. The phototropic nature of nauplii caused them to migrate in the direction of light toward the bottom of the tube, facilitating their separation from unhatched cysts. First, a stock solution of DlyL was made by dissolving the lectin in artificial seawater in a concentration of 200 µg/mL. This assay was made in Linbro plates (24-well) where each well contained 10 *Artemia* nauplii in a final volume of 2 mL. Stock solution of the lectin was then added to the wells at final concentrations of 6.25, 12.5, 25, 50 and 100 µg/mL. The experiments were performed in triplicate, and negative control was the artificial water and the *Artemia* nauplii in the absence of the lectin. After 24 h, the number of dead nauplii in each well was counted. In order to verify the importance of the carbohydrate binding activity in the toxicity caused by DlyL, the lectin was incubated with 100 mM d-mannose, and the *Artemia* lethality test was made at final concentration of 100 µg of lectin by mL of artificial seawater.

### 3.10. Determination of the LC_50_ Value

Data obtained from the toxicity assay were plotted in a simple program (Microsoft Excel 2013) on a personal computer. The LC_50_ values were computerized from the percentage of death and logarithm concentrations by probit analysis.

### 3.11. DlyL Immobilization on Sepharose 4B

Cyanogen bromide-activated Sepharose 4B was used for DlyL immobilization [35]. CNBr-activated Sepharose 4B (0.5 g) was washed with HCl 1 mM, followed by 100 mM NaHCO_3_ in 0.5 M NaCl, pH 8.3. Incubation (1 h, 25 °C) was performed with DlyL (15 mg of protein). After filtration and washing with NaHCO_3_ solution, the DlyL-Sepharose 4B matrix was washed with 100 mM Tris-HCl in 0.5 M pH 8.0, followed by 100 mM sodium acetate in 0.5 M NaCl at pH 4.0. DlyL retention was determined by calculating the difference between the weight of loaded DlyL and the amount of protein found before washing. To analyze the performance of immobilization and capacity of DlyL-Sepharose 4B matrix to purify glycoproteins, trials were carried out with ovalbumin. Ovalbumin (2 mg) was chromatographed on DlyL-Sepharose 4B column (0.8 × 2.7 cm) equilibrated with 150 mM NaCl plus 5 mM CaCl_2_ and 5 mM MnCl_2_ (flow rate of 0.33 mL/min). The lectin affinity support was then washed with the same buffer, and bound proteins were then eluted with buffer containing 0.2 M α-methyl-d-mannoside. Eluted fractions were monitored by absorbance at 280 nm. 

## 4. Conclusions

DlyL is a mannose/glucose-binding lectin purified from the protein extract of the seeds of *Dioclea lasiophylla* and further characterized physicochemically. Similar to other Diocleinae lectins, DlyL has an α-chain, which generates two smaller “fragment” chains, β and γ, as a consequence of post-translational circular permutation, a posttranslational modification typical of Diocleinae lectins. In addition, DlyL was demonstrated to have low toxicity against *Artemia* sp. nauplii with dose-dependent effect, and immobilized DlyL showed high potential as a tool for purification of glycoproteins, enabling its use in glycoproteomics studies.
